# Is there an inverted-U relationship between creativity and psychopathology?

**DOI:** 10.3389/fpsyg.2014.00750

**Published:** 2014-07-28

**Authors:** Anna Abraham

**Affiliations:** Department of Community Medicine and Behavioural Sciences, Faculty of Medicine, Kuwait UniversityJabriya, Kuwait

**Keywords:** top down control, information processing, creative cognition, mental illness, divergent thinking, psychopathology, creativity, inverted-U function

**A commentary on the Research Topic**

**Madness and Creativity: Yes, No or Maybe?**

Edited by Anna Abraham

Few issues polarize the scientific community within the field of creativity as the purported association between creativity and psychopathology. The idea that the two are intimately linked dates back to Greek antiquity where the mental state of creative individuals during idea generation was noted to be highly aberrant. However, such eccentric states were not held to reflect clinical levels of mental illness until the 1800s (Becker, 2001).

The intuitive appeal of this connection partly stems from the commonalities we associate with mental illness and creativity, including a high tolerance for ambiguity, the ability to generate non-generic conceptual connections, and the adoption of alternative perspectives (Abraham, in press). Moreover, higher than average incidences of mental illness are found among people who practice professions that demand high levels of creativity, such as visual artists and writers (Kyaga et al., 2011; Simonton, 2014). The information processing mechanism that is generally proposed as underlying the link between creativity and psychopathology is that shortcomings during normative cognition (e.g., cognitive disinhibition), that are characteristic of certain psychiatric populations (e.g., psychosis), may translate to benefits in the context of creative cognition (Carson, 2011).

There are, however, also good grounds to be skeptical of the “mad genius” meme, which some argue is a quixotic notion at best (Schlesinger, 2009). For one thing, many of the studies that have been used to support this idea have come under a lot of criticism on methodological counts (Thys et al., 2014). Some have even shown that the presence of psychopathological traits explains only a paltry amount of the variance in creative performance (Silvia and Kimbrel, 2010). In addition, notwithstanding notable exceptions (e.g., van Gogh), individuals who achieved creative eminence in their fields were not operating at peak levels of productivity when they reached the point of severe mental illness.

So how can we make sense of this picture given that the evidence of a positive relationship between creativity and mental illness is clearly mixed? One approach would be to breakdown the empirical investigations that have assessed this link into meaningful categories based on a specific criterion and to evaluate whether any systematic patterns emerge as a result.

The madness-creativity link has, for instance, been investigated by assessing the performance of both psychiatric populations as well as subclinical populations on measures of creativity (Kaufman, 2014). The most well studied psychiatric populations in this regard include individuals with schizophrenia, bipolar disorder, attention deficit hyperactivity disorder (ADHD) and autism. Subclinical populations have also been widely assessed, and these refer to high risk healthy populations who are defined as such because they exhibit a high degree of mental illness-relevant personality traits. The rationale behind investigating subclinical groups is that studying high-functioning individuals who show some degree of predisposition for a clinical disorder enables us to understand the workings of the information processing biases related to that disorder without the burden of having to control for variables that can exert a confounding effect in studies on clinical populations (e.g., medication). Indeed, much evidence points to similarities in the information processing biases (e.g., latent disinhibition) typical of specific clinical groups (e.g., schizophrenia) and their respective subclinical populations (e.g., high psychoticism or schizotypal groups).

One means by which the creativity-psychopathology link can be investigated then is to focus on investigations of populations that are documented to have similar information processing biases and to cluster these studies by the type of population (clinical/subclinical) and the severity of disorder (high/low dysfunction). Let's take the premise that reduced top-down down control (influence of knowledge and expectations) on information processing can have a facilitative or debilitative effect on creative cognition. A number of psychiatric populations, such as ADHD and schizophrenia, are associated with poor top-down control and corresponding fronto-striatal dysfunction (Bradshaw and Sheppard, 2000), but these vary greatly in terms of severity. ADHD is associated with top-down deficits such as high levels of distractibility, impulsivity and poor inhibitory control functioning. But these are mild relative to those typically associated with schizophrenia within domains like executive function, working memory, inhibitory control and fluency. Milder still are negative biases in top-down control, such as latent disinhibition, that have been reported in subclinical groups. So does any viable pattern emerge when clustering the findings of such behavioral and neuropsychological studies according to the degree and/or type of top-down insufficiencies: clinical-severe, clinical-moderate, and subclinical-mild?

A number of studies on subclinical-mild populations, such as individuals who are characterized by the presence of a high degree of either schizotypal or psychoticism traits, have demonstrated that they consistently perform better than their low trait counterparts on some measures of creativity (Schuldberg, 2005; Acar and Sen, 2013). The same is true of populations who display clinical-moderate levels of top-down dysfunction, such as ADHD (Abraham et al., 2006; Healey and Rucklidge, 2006). In contrast, populations who are characterized by clinical-severe levels of top-down dysfunction, such as schizophrenia, perform poorly on almost all measures of creativity (Abraham et al., 2007; Jaracz et al., 2012). This pattern of findings suggests that while subclinical-mild and clinical-moderate levels of top-down dysfunction can, under specific conditions, confer selective advantages in creative cognition, clinical-severe levels of top-down dysfunction leads to impoverished creative thinking. A minimal level of function is probably essential to develop the original ideas one generates into something more tangible than a fleeting thought.

The effects of alterations in top-down control on creative performance can therefore be parsimoniously conceptualized in terms of an inverted-U shaped function or an inverted backward-J function (Figure 1). Direct investigations are necessary to reveal the precise pattern of this relationship. While diffuse or defocused top-down control in information processing may abet creative cognition, too much (normal) or too little (defective) top-down control can hinder or disrupt the same (Abraham, in press). An inverted-U function in this context is postulated to account for the abundance of conflicting findings associated with investigating the creativity-psychopathology link. The strength of this hypothesis is that it is one that readily lends itself to empirical investigation.

**Figure 1:**
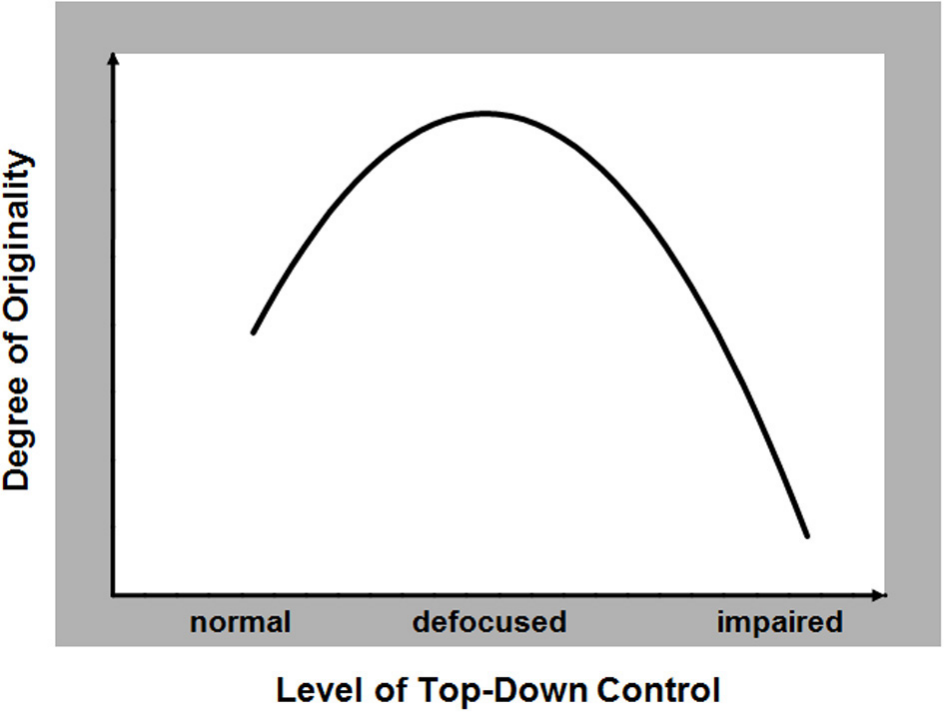
**The hypothesized relationship between the capacity to generate original responses during creative thinking (range: low to high) and the degree of functionality in top-down control of information processing (range: normal to impaired)**.

## Conflict of interest statement

The author declares that the research was conducted in the absence of any commercial or financial relationships that could be construed as a potential conflict of interest.
